# Non-invasive Assessment of Mitochondrial Oxygen Metabolism in the Critically Ill Patient Using the Protoporphyrin IX-Triplet State Lifetime Technique—A Feasibility Study

**DOI:** 10.3389/fimmu.2020.00757

**Published:** 2020-05-07

**Authors:** Charles Neu, Philipp Baumbach, Alina K. Plooij, Kornel Skitek, Juliane Götze, Christian von Loeffelholz, Christiane Schmidt-Winter, Sina M. Coldewey

**Affiliations:** ^1^Department of Anesthesiology and Intensive Care Medicine, Jena University Hospital, Jena, Germany; ^2^Septomics Research Center, Jena University Hospital, Jena, Germany; ^3^Center for Sepsis Control and Care, Jena University Hospital, Jena, Germany

**Keywords:** sepsis, COMET, mitochondrial dysfunction, critically ill patients, protoporphyrin IX-triplet state lifetime technique, cellular oxygen metabolism, mitochondrial oxygen metabolism, mitochondrial oxygen tension

## Abstract

The imbalance of oxygen delivery and oxygen consumption resulting in insufficient tissue oxygenation is pathognomonic for all forms of shock. Mitochondrial function plays an important role in the cellular oxygen metabolism and has been shown to impact a variety of diseases in the intensive care setting, specifically sepsis. Clinical assessment of tissue oxygenation and mitochondrial function remains elusive. The *in vivo* protoporphyrin IX-triplet state lifetime technique (PpIX-TSLT) allows the direct, non-invasive measurement of mitochondrial oxygen tension (mitoPO_2_) in the human skin. Our recently established measurement protocol for the Cellular Oxygen Metabolism (COMET) Monitor, a novel device employing the PpIX-TSLT, additionally allows the evaluation of oxygen consumption (mitoVO_2_) and delivery (mitoDO_2_). In the intensive care setting, these variables might provide new insight into mitochondrial oxygen metabolism and especially mitoDO_2_ might be a surrogate parameter of microcirculatory function. However, the feasibility of the PpIX-TSLT in critically ill patients has not been analyzed systematically. In this interim study analysis, we evaluated PpIX-TSLT measurements of 40 patients during the acute phase of sepsis. We assessed (a) potential adverse side effects of the method, (b) the rate of analyzable measurements, (c) the stability of mitoPO_2_, mitoVO_2_, and mitoDO_2_, and (d) potential covariates. Due to excessive edema in patients with sepsis, we specifically analyzed the association of patients' hydration status, assessed by bioimpedance analysis (BIA), with the aforementioned variables. We observed no side effects and acquired analyzable measurements sessions in 92.5% of patients (*n* = 37/40). Different measures of stability indicated moderate to good repeatability of the PpIX-TSLT variables within one session of multiple measurements. The determined limits of agreement and minimum detectable differences may be helpful in identifying outlier measurements. In conjunction with signal quality they mark a first step in developing a previously unavailable standardized measurement quality protocol. Notably, higher levels of hydration were associated with lower mitochondrial oxygen tension. We conclude that COMET measurements are viable in patients with sepsis. To validate the clinical and diagnostic relevance of the PpIX-TSLT using the COMET in the intensive care setting, future studies in critically ill patients and healthy controls are needed.

## Introduction

Sepsis is defined as a life-threatening host response toward infection resulting in organ dysfunction (1). Despite advances in the pathophysiological understanding of this condition, research has not led to significant changes in sepsis therapy. It therefore remains one of the most prevalent critical conditions worldwide with only supportive therapy. A major hallmark of sepsis and septic shock in particular is disturbed tissue oxygenation. Hitherto, surrogate parameters of tissue oxygenation have been shown to be of limited reliability. Early goal-directed therapy, focusing on central venous oxygen saturation as a surrogate parameter of tissue oxygenation, for example, has shown no overall benefit in a recent meta-analysis (2) leaving clinicians without any evidence on which to base their decisions. Therefore, there is a need for new research into the direct measurement of tissue oxygenation.

In recent years, the mitochondrion has become a focus of medical research. The mitochondrion, as “powerhouse” of the cell, is responsible for the regulation of cellular oxygen metabolism and could therefore pose a potential target for the measurement of tissue oxygenation. Studies have demonstrated a pathophysiological involvement of mitochondrial function in, among others, cancer, heart, and age-related diseases (3–5). In sepsis, studies have yielded first indications of a dysregulated mitochondrial function. Clinical trials showed increased mitochondrial protein synthesis in patients with sepsis (6, 7). Another study demonstrated a decreased mitochondrial function in biopsies from patients with sepsis (8). Thus far, clinical measurements were performed in muscle biopsies. As the analysis is *ex vivo* there may be pre-analytical confounders influencing the results. Also, the procedure is invasive and therefore unlikely to be used routinely for diagnostic purposes. Non-invasive direct measurements of mitochondrial function in patients could pose a feasible method to assess tissue oxygenation in patients with sepsis.

Mik et al. introduced the protoporphyrin IX-triplet state lifetime technique (PpIX-TSLT) for non-invasively measuring mitochondrial oxygen tension (mitoPO_2_) (9). In brief, the method is based on the delayed fluorescence of protoporphyrin IX (PpIX), the naturally occurring precursor of the heme molecule, which can be enriched in skin cells by external application of 5-aminolevulinic acid (5-ALA). The delayed fluorescence is induced by pulses of green light and is inversely correlated with mitochondrial oxygen tension. The recent development of the CE-certified Cellular Oxygen METabolism Monitor (COMET) now enables the application of the PpIX-TSLT in the clinical setting. As this device allows the direct measurement of oxygen metabolism on the cellular level, it could be employed as a diagnostic tool for patients with sepsis. Thus far, the COMET has been employed in a pharmacological study (10) and in healthy controls (11, 12).

The primary objective of this study was to test how feasible the PpIX-TSLT measurements are in the acute phase of sepsis during the treatment at the critical care unit. The secondary objectives were to assess the distribution and the stability of PpIX-TSLT variables for single measurements and to identify potential covariates, in particular the patients' fluid status.

## Methods

### Patient Sample

This study is an intermediate analysis of patients from the study *Identification of cardiovascular and molecular prognostic factors for the medium- and long-term outcomes of sepsis* (ICROS, DRKS00013347, and NCT03620409). Patients with sepsis were recruited at the intensive care units of the Jena University Hospital. The inclusion and exclusion criteria of the study are presented in Table 1. For PpIX-TSLT measurements, contraindications were: allergies to contents of the Alacare® plaster (photonamic, Wedel, Germany), porphyria, skin conditions aggravated by sunlight, or increased sensitivity to light. For BIA measurements contraindications were: electronic implants (e.g., pacemaker) or active prostheses. Study physicians obtained written informed consent from either the patient or the patient's legal proxy if the patient was incapacitated. The study was approved by the ethics committee of the Friedrich Schiller University Jena (5276-09/17).

**Table 1:** Inclusion and exclusion criteria for the study.

**Inclusion criteria**
Sepsis or septic shock meeting Sepsis-3 criteria (1)
Onset of first infection-caused organ dysfunction no longer than 72 h before enrolment
At least 18 years of age
Written informed consent from the patient, legal representative or proxy, or preliminary consent after consultation of an independent medical doctor
**Exclusion criteria**
Cardiac surgery in the last 12 months
Significant heart disease:
Endocarditis
Higher degree valve disorders (severe valvular heart disease, symptomatic aortic valve stenosis, moderate mitral regurgitation with reduced ejection fraction)
Congenital heart defect (e.g., transposition of the great arteries, tetralogy of Fallot, atrioventricular septal defect)
Hemodynamically relevant shunting heart defect
Reduced cardiac output (EF < 45% or 10% below norm^*^) prior to sepsis
Pulmonary arterial hypertension prior to onset of sepsis
Myocardial infarction 12 months prior to onset of sepsis
History of heart transplantation
Cardiopulmonary resuscitation 4 weeks prior to onset of sepsis
History of pneumonectomy
Child C liver cirrhosis
Contraindications for transesophageal echocardiography and insufficient quality of transthoracic echocardiography
Terminal chronic kidney disease with dialysis
Sepsis within 8 months prior to onset of sepsis
Pregnancy/breast-feeding
Therapy limitation/do-not-resuscitate order
Remaining life expectancy <6 months due to other causes than sepsis
Prior participation in this study
Participation in another interventional study

* *Left ventricular ejection fraction >52% in men, >54% in women according to the American Society of Echocardiography and the European Association of Cardiovascular Imaging (13)*.

### PpIX-TSLT Measurements

**Figure 1:**
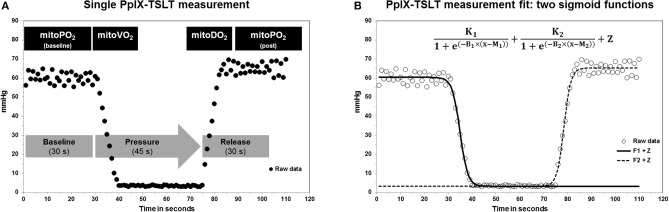
**(A)** Description of a single PpIX-TSLT measurement to obtain mitochondrial oxygen tension (mitoPO_2_), mitochondrial oxygen consumption (mitoVO_2_), and mitochondrial oxygen delivery (mitoDO_2_). **(B)** Illustration of the raw PpIX-TSLT data and the two sigmoid fit functions to estimate the PpIX-TSLT variables.

PpIX-TSLT measurements took place within 3 ± 1 days after the onset of sepsis. A 4 cm^2^ patch containing 5-ALA (Alacare®, photonamic, Wedel, Germany) was applied to the clavipectoral triangle at least 5 h before the planned measurement to ensure sufficient accumulation of PpIX. Before application, the skin was cleaned and prepared with an abrasive paste (skinPure®, Nihon Kohden, Rosbach, Germany). For 48 h after application, the skin was protected from light with an additional patch. The measurements were performed with the COMET measurement system (Photonics Healthcare, Utrecht, Netherlands). Before the measurement session, the sensor was shielded from light and applied to the prepared skin. During one session multiple measurements with the following parameters were performed (see also Figure 1): In the first 30 s, the mitoPO_2 : baseline_ was measured. Thereafter, we applied pressure to the sensor for 45 s to inhibit the microcirculation of that part of the skin and to measure oxygen consumption (mitoVO_2 : maximum_ and mitoVO_2 : average_). Finally, the pressure was released to evaluate the re-oxygenation (mitoDO_2 : maximum_ and mitoDO_2 : average_) and post-re-oxygenation mitoPO_2_ (mitoPO_2 : post_). A single measurement took 105 s and was performed at least three times per session. We used two complementary sigmoid functions to fit the raw PpIX-TSLT signals of each single measurement:

(1) $$\begin{array}{l} \frac{K_{1}}{1+e^{\left(-B_{1}\times \left(x-M_{1}\right)\right)}}+\frac{K_{2}}{1+e^{\left(-B_{2}\times \left(x-M_{2}\right)\right)}}+Z \end{array}$$

The estimation of these function parameters allows a direct inference of the PpIX-TSLT variables. For details please [see (12)].

We used a self-developed program (Halley) under MATLAB (MATLAB and Statistics Toolbox Release 2017a, The MathWorks, Inc., Natick, Massachusetts, United States) for data management, data preparation and PpIX-TSLT variable estimation.

### Bioimpedance Vector Analysis

The patient's fluid status was evaluated using the seca medical Body Composition Analyzer 525 (seca Germany, Hamburg, Germany). For bioimpedance vector analysis (BIVA), raw impedance variables, resistance (R) and reactance (Xc), were standardized to body height (R_/height_, Xc_/height_) in meters (14). Two characteristics of the resulting bivariate vector were analyzed: the phase angle [arc tangent of (Xc/R) × 180°/π] as vector orientation and the vector length (square root of ${\text{Xc}}_{\text{height}}^{2}$ + ${\text{R}}_{\text{height}}^{2}$). Especially the latter one is an indicator of the hydration status (14, 15) and has already been proven to be applicable in the critical care setting (16). Short vectors, resulting from decreased resistance, indicate high levels of hydration and can indicate the presence of edema secondary to low oncotic pressure or endothelial barrier dysfunction. Long vectors, resulting from increased resistance, are an indicator of a low hydration status, i.e., dehydration. Where feasible, body height, and weight were obtained by means of a measuring tape and the Seca bed scale 985 (Seca Germany, Hamburg, Germany), respectively. Otherwise, information from the medical history was used.

### Descriptive Analysis

In descriptive analysis, means, standard deviations (SD), medians, as well as first and third quartiles (Q_1_/Q_3_) are reported. For categorical and dichotomous variables we report absolute and relative frequencies. The distribution of the PpIX-TSLT variables was assessed using histograms, Q-Q-plots, Shapiro-Wilk-Tests, and an estimation of kurtosis and skewness with corresponding standard errors (SE) and kurtosis excess (kurtosis – 3).

### Stability of PpIX-TSLT Variables

To analyze the repeatability, i.e., stability, of PpIX-TSLT variables during one measurement session we took the following steps: First, descriptive mean differences between measurement pairs and corresponding *p*-values of the paired samples *t*-tests are reported. Second, Pearson correlation coefficients of all available measurement pairs are presented. Correlation coefficients can be interpreted in the following way: *r* < 0.10 negligible, *r* 0.1–0.39 weak, 0.40–0.69 moderate, 0.70–0.89 strong, 0.90–1.00 very strong (17). Third, measurement pairs were analyzed using Bland-Altman plots to assess limits of agreement (LOA) (18, 19). In detail, the mean of a measurement pair is plotted against the difference between both single measurements (LOA = mean of the differences ± 1.96 × SD of the differences). The corresponding 95% confidence intervals (95%CI) for LOA were obtained using two-sided tolerance factors (20). At population level, 95% of PpIX-TSLT variable differences between single PpIX-TSLT measurements within one session, according to our protocol, should lie within these LOA. Fourth, intra-class correlation coefficients (ICC) for all available measurement pairs using the two-way mixed effects analysis of variance (ANOVA) for single measures with absolute agreement were obtained (21). ICCs and corresponding confidence intervals can be interpreted as follows: ICC < 0.5 poor, 0.5 ≤ ICC ≤ 0.75 moderate, 0.75 ≤ ICC ≤ 0.90 good, and ICC > 0.90 excellent reliability (21). Finally, the standard error of measurement (SEM) as the square root of the mean square error term from repeated-measures ANOVA for all available measurement pairs (22) and the Minimum Detectable Difference (MDD, SEM × 1.96 × 2^1/2^) are reported.

### Exploratory Analysis of Potential Covariates

Potential covariates for PpIX-TSLT variables were analyzed parametrically and non-parametrically with Pearson correlation coefficients and Spearman's rank correlation coefficients, respectively. PpIX-TSLT variables from multiple single measurements were averaged before correlative analysis. Results were additionally visualized with scatterplots and regression lines from the simple linear regression models (PpIX-TSLT variables served as dependent variables). We considered the following variables: sex, age, BIVA variables (see above), PpIX-TSLT-associated variables (duration of 5-ALA application, average signal quality, room, sensor, skin, and body temperature, goodness of fit of the fitting procedure), physiological data (heart rate, systolic and diastolic blood pressure, SpO_2_, hemoglobin, fluid balance), and treatment-associated data (initial SOFA score, catecholamine status, ventilation status). Treatment-related variables were obtained from electronic patient records on ICU (Copra System, Berlin, Germany).

For statistical analysis we used SPSS Statistics 24 (IBM Corporation, Armonk, NY, USA) and R [Version 3.5.1, Vienna, Austria (23)]. We applied a significance level of 5% and report two-sided *p*-values.

## Results

### Sample Description

**Figure 2:**
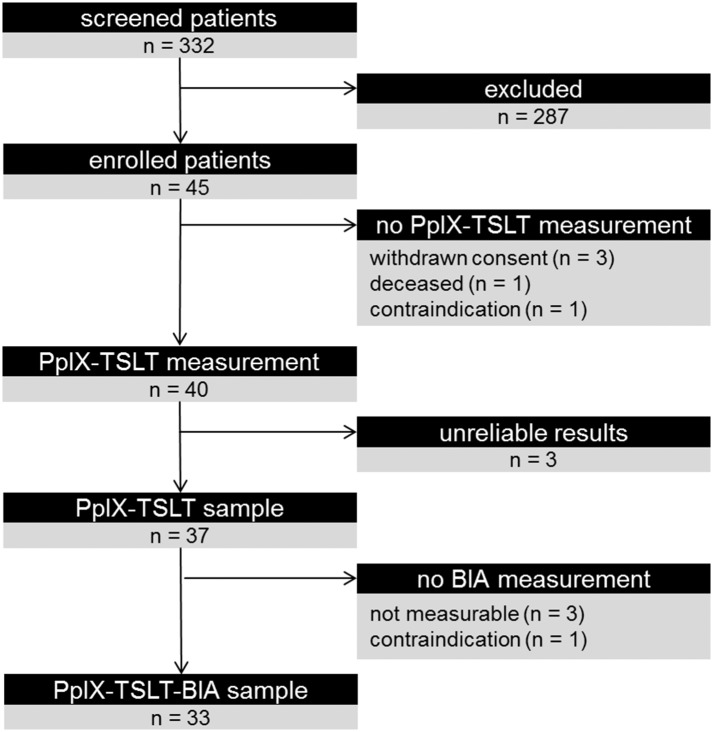
Overview of the patient inclusion and analytical cohorts. Results for primary analysis are reported from the PpIX-TSLT sample. Results for Bioimpedance Analysis (BIA) and additional correlative analyses with PpIX-TSLT variables are reported for the PpIX-TSLT-BIA sample.

Figure 2 summarizes the study inclusion. Of 332 screened patients, 45 patients with sepsis were enrolled in the study. After excluding patients who withdrew consent, died before the measurement or had contraindications, PpIX-TSLT measurements were performed on 40 patients. 37 patients with reliable PpIX-TSLT measurements were included in the primary analysis. Demographic and clinical characteristics of the study sample are displayed in Table 2.

**Table 2:** Demographics and clinical characteristics (*n* = 37 patients).

**Variable**	**[Unit]**	**Median**	**Q_**1**_**	**Q_**3**_**
Age	[years]	70.00	58.00	79.00
Initial SOFA increase	[points]	5.00	4.00	7.00
Body weight	[kilogram]	76.20	68.00	92.00
Body height	[meter]	1.72	1.65	1.75
BMI	[kg/m^2^]	27.13	23.57	31.02
Body surface	[m^2^]	1.89	1.66	2.04
**Variable**	**Category**		***n***	**%**
Sex	Female		13	35.1
	Male		24	64.9
Sepsis focus	Pneumonia		17	45.9
	Intra-abdominal/ gastrointestinal		13	35.1
	Thoracic		2	5.4
	Urogenital		3	8.1
	Bone/soft-tissue		1	2.7
	Skin		1	2.7
Ventilation^a^	Spontaneous		20	54.1
	Invasive/non-invasive		17	45.9
Catecholamines^b^	None		11	29.7
	Medium^b^		11	29.7
	High^c^		15	40.5

*For continuous measures median, first and third quartile (Q_1_/Q_3_) are displayed. For categorical variables number (n) and percent (%) are shown*.

a *Any duration of invasive ventilation. Non-invasive ventilation > 6 h*.

b *Dopamine > 5 μg/kg/min or epinephrine ≤ 0.1 μg/kg/min or norepinephrine ≤ 0.1 μg/kg/min*.

c *Dopamine > 15 μg/kg/min or epinephrine > 0.1 μg/kg/min or norepinephrine > 0.1 μg/kg/min*.

### Primary Analysis

In 37 (92.5%) of the 40 included patients with sepsis, reliable PpIX-TSLT measurement sessions could be recorded. In the 3 patients with unreliable measurement sessions either the signal quality was too low and/or mitoVO_2_ could not be induced consistently during multiple measurements. We did not observe any side effects of the 5-ALA application or the PpIX-TSLT measurement itself in any patient.

### Secondary Objectives

#### Descriptive Statistics and Variable Distribution

The descriptive statistics of the PpIX-TSLT variables are displayed in Table 3. Additional information on variable distribution is provided in Supplement S-1. Shapiro-Wilk tests indicated that mitoPO_2_ variables were normally distributed (*p* > 0.05). The *p*-values of the Shapiro-Wilk tests for the other PpIX-TSLT variables were significant (*p* < 0.01). All PpIX-TSLT parameters were positively skewed and kurtosis excess values ranged between −2.4 (mitoPO_2 : baseline_, platykurtic) to 3.0 (mitoVO_2 : average_, leptokurtic). After removing outlier values (values >Q_3_ + 1.5 times the interquartile range, see also Table 3) none of the *p*-values of the Shapiro-Wilk tests reached significance (*p* > 0.05) indicating normally distributed values.

**Table 3:** Descriptive statistics for PpIX-TSLT variables.

**Variable**	**[Unit]**	***n***	**Mean ± SD**	**[95%CI]**	**Median**	**Q_**1**_**	**Q_**3**_**	**Threshold**	
								**Lower**	**Upper**
MitoPO_2 : baseline_	[mmHg]	37	61.86 ± 19.97	[55.42, 68.29]	65.15	49.21	70.84	16.75	103.30
MitoPO_2 : post_	[mmHg]	37	55.52 ± 15.64	[50.48, 60.56]	56.75	44.18	64.38	13.88	94.68
MitoVO_2 : maximum_	[mmHg/s]	37	4.82 ± 2.39	[4.05, 5.59]	4.65	3.16	5.71	0.00	9.54
MitoVO_2 : average_	[mmHg/s]	37	3.43 ± 1.71	[2.88, 3.98]	3.31	2.25	4.07	0.00	6.80
MitoDO_2 : maximum_	[mmHg/s]	37	6.87 ± 4.06	[5.56, 8.18]	5.76	4.23	7.56	0.00	12.57
MitoDO_2 : average_	[mmHg/s]	37	4.89 ± 2.89	[3.96, 5.82]	4.11	3.01	5.39	0.00	8.95

*All PpIX-TSLT variables from one measurement session were averaged before descriptive analysis*.

*mitoPO_2_, mitochondrial oxygen tension; mitoVO_2_, mitochondrial oxygen consumption; mitoDO_2_, mitochondrial oxygen delivery*.

*Thresholds are defined as Q_1_ – 1.5 × interquartile range and Q_3_ + 1.5 × interquartile range*.

*SD, standard deviation; 95%CI, 95 percent confidence interval, Q_1_/Q_3_, first and third quartile*.

#### Stability of PpIX-TSLT Measurements

MitoPO_2 : baseline_ values showed a mean difference of 4.42 mmHg between subsequent measurements, resulting in a significant *p*-value of the corresponding paired *t*-test (Table 4). In addition, both mitoDO_2_ variables differed significantly between subsequent measurements (*p* < 0.01) with a mean difference of 1.07 (mitoDO_2 : maximum_) and 0.76 (mitoDO_2 : average_), respectively. MitoPO_2 : Post_ and both mitoVO_2_ variables did not differ significantly between subsequent measurements. All variables were moderately (mitoDO_2_) to strongly (all others) correlated between subsequent measurements (Table 4). The limits of agreement and corresponding Bland-Altman-Plots for subsequent measurements are displayed in Table 4 and Supplement S-2. The intraclass correlation coefficients for all PpIX-TSLT variables showed significant *p*-values (*p* < 0.001) and ranged between 0.652 (moderate) for mitoDO_2_ variables and 0.805 (good) for mitoVO_2_ variables (Table 5). Finally, the standard error of measurement (SEM) and Minimum Detectable Difference (MDD) of PpIX-TSLT variables are displayed in Table 5.

**Table 4:** Intra-session stability of the PpIX-TSLT variables (*n* = 36 patients with at least 2 reliable PpIX-TSLT measurements).

			**Difference**								**Limits of agreement (LOA)**					
**Variable**	**[Unit]**	***n***	**Δmean**		**SD**	**[95%CI]**	***p*_***ttest***_**	***r***	***p*_***r***_**	**Lower**	**95%CI**		**Upper**	**95%CI**	
MitoPO_2 : baseline_	[mmHg]	114	−4.42	±	13.62	[−6.92, −1.92]	**0.001**	0.75	**<0.001**	−31.12	−35.27	−28.14	22.28	19.30	26.43
MitoPO_2 : post_	[mmHg]	114	−0.95	±	11.24	[−3.01, 1.11]	0.369	0.72	**<0.001**	−22.99	−26.41	−20.53	21.09	18.63	24.51
MitoVO_2 : maximum_	[mmHg/s]	114	−0.02	±	1.54	[−0.30, 0.26]	0.901	0.81	**<0.001**	−3.03	−3.50	−2.70	3.00	2.66	3.47
MitoVO_2 : average_	[mmHg/s]	114	−0.01	±	1.10	[−0.22, 0.19]	0.893	0.81	**<0.001**	−2.16	−2.50	−1.92	2.14	1.90	2.47
MitoDO_2 : maximum_	[mmHg/s]	114	1.07	±	3.96	[0.34, 1.79]	**0.005**	0.68	**<0.001**	−6.69	−7.89	−5.82	8.82	7.96	10.03
MitoDO_2 : average_	[mmHg/s]	114	0.76	±	2.82	[0.24, 1.28]	**0.005**	0.68	**<0.001**	−4.76	−5.62	−4.14	6.28	5.66	7.14

*Mean difference (Δmean), corresponding standard deviation (SD), and 95% confidence intervals (95%CI) for PpIX-TSLT measurement pairs are shown. Additionally, Pearson correlation coefficients (r), corresponding p-values (p_r_), and p-values of the paired t-tests (p_ttest_) for PpIX-TSLT measurement pairs are displayed. Further, limits of agreement (LOA) with corresponding 95%CI and p-values are shown. P < 0.05 are printed in bold*.

**Table 5:** Intraclass correlation coefficients (ICC) with corresponding 95% confidence intervals, standard error of measurement (SEM), and minimum detectable difference (MDD) for PpIX-TSLT variables (*n* = 36 patients with at least 2 reliable PpIX-TSLT measurements).

					**Intraclass correlation coefficients**			
**Variable**	***n***	**SEM**	**MDD**	**[Unit]**	**ICC**	**95%CI**		***p***
MitoPO_2 : baseline_	114	9.63	26.70	[mmHg]	0.729	0.612	0.811	**<0.001**
MitoPO_2 : post_	114	7.95	22.04	[mmHg]	0.717	0.615	0.796	**<0.001**
MitoVO_2 : maximum_	114	1.09	3.01	[mmHg/s]	0.805	0.729	0.861	**<0.001**
MitoVO_2 : average_	114	0.78	2.15	[mmHg/s]	0.805	0.729	0.861	**<0.001**
MitoDO_2 : maximum_	114	2.80	7.75	[mmHg/s]	0.652	0.526	0.749	**<0.001**
MitoDO_2 : average_	114	1.99	5.52	[mmHg/s]	0.652	0.526	0.749	**<0.001**

*P < 0.05 are printed in bold*.

*ICC < 0.5 poor, 0.5 ≤ ICC ≤ 0.75 moderate, 0.75 ≤ ICC ≤ 0.90 good, ICC > 0.90 excellent (21)*.

#### Correlative Analysis

Statistically significant associations between PpIX-TSLT variables and tested covariates are shown in Table 6. The descriptive statistics for BIVA-variables and potential covariates are shown in Supplement S-3. In summary, mitoPO_2 : baseline_ correlated positively with height-standardized vector length of BIVA. Maximum and average mitoVO_2_-variables showed correlation coefficients of 1. The same applies for mitoDO_2_ variables. For this reason, only results for mitoVO_2 : average_ and mitoDO_2 : average_ are reported. MitoVO_2_ variables were positively correlated with the goodness of fit (*R*^2^) of the fitting function. MitoDO_2_ variables were negatively correlated with the duration of 5-ALA application, the average signal quality during PpIX-TSLT measurement and body height. In addition, mitoDO_2_ variables were positively correlated with the room temperature. Finally, the mean signal quality of one measurement was positively correlated with the duration of the 5-ALA application and negatively correlated with the room temperature. All other tested covariates showed no statistically significant correlation coefficients with PpIX-TSLT variables (data not shown). MitoPO_2 : baseline_ tended to differ between female (median: 69.95, Q_1|3_: 54.03 | 74.08) and male patients (median: 57.19, Q_1|3_: 44.36 | 69.25). In addition, we found significant sex differences between mitoDO_2 : average_ with higher values for females (median: 5.25, Q_1|3_: 3.45 | 9.98) compared to males (median: 3.73, Q_1|3_: 2.55 | 5.27, *U* = 84, *p* = 0.022). Neither the ventilation status nor the catecholamine dosage was significantly associated with any of the PpIX-TSLT variables. After adjusting for multiple testing using the Bonferroni-Holm method, only the *p*-value for the association between mitoVO_2_ variables and the goodness of fit (*R*^2^) reached significance (adjusted *p*-values not shown).

**Table 6:** Main findings of the correlation analyses for the PpIX-TSLT variables and potential covariates.

**Variable**	**Covariate**	***n***	**ρ**	***p*_**ρ**_**	***r***	***p*_***r***_**
MitoPO_2 : baseline_	BIVA: vector length^†^	33	0.36	**0.042**	0.23	0.191
MitoVO_2 : average_	Goodness of Fit	37	0.71	**<0.001**	0.58	**<0.001**
MitoDO_2 : average_	Body height	37	−0.38	**0.020**	−0.32	0.051
	Duration of 5-ALA application	36	−0.37	**0.024**	−0.37	**0.026**
	Signal Quality	37	−0.34	**0.039**	−0.27	0.113
	Temperature: room	37	0.40	**0.015**	0.25	0.140
Signal quality	Duration of 5-ALA application	36	0.35	**0.038**	0.22	0.198
	Temperature: room	37	−0.34	**0.039**	−0.39	**0.016**

*Spearman's rank correlation coefficient (ρ) with the corresponding p-value (p_**ρ**_) and the Pearson correlation coefficient (r) with the corresponding p-value (p_r_) are shown. P < 0.05 are printed in bold*.

*mitoPO_2_, mitochondrial oxygen tension; mitoVO_2_, mitochondrial oxygen consumption; mitoDO_2_, mitochondrial oxygen delivery*.

† *Obtained from height-standardized resistance and reactance values*.

## Discussion

### Feasibility of PpIX-TSLT Measurements of Mitochondrial Function in Patients With Sepsis

In this study, we report for the first time direct *in vivo* assessment of mitochondrial oxygen metabolism by PpIX-TSLT measurements in a cohort of patients with sepsis. Thus far, only reports in healthy subjects or surgical patients have been published. In our cohort of 40 patients, PpIX-TSLT measurements yielded analyzable datasets in 92.5% of patients. Also, no side effects of the measurements were observed. We therefore conclude that PpIX-TSLT measurements with the COMET are feasible in patients with sepsis in the ICU setting. In our previous study, analyzable results were obtained from 75% of healthy subjects (12). We believed at the time that compliance problems concerning 5-ALA application may have contributed to this relatively low success rate. As the ICU offers a standardized environment, protocol adherence was very high. Hence, our results may confirm our assumption and stress the importance of controlling the duration of 5-ALA application. Of the three measurements that failed in this study, one was due to low signal quality. This may have been due to inadequate skin absorption of 5-ALA despite using a standardized protocol. In the other two cases, mitoVO_2_ could not be induced. This phenomenon is not entirely understood and deserves future attention.

### Distribution and Stability of PpIX-TSLT Variables

After removing outliers, all variables were normally or near-to-normally distributed. In our previous trial, PpIX-TSLT variables were also distributed near to normal (12). Therefore, we conclude both parametric and non-parametric analyses may be applicable for PpIX-TSLT variables.

All variables were moderately to highly correlated between replicate measurements. All ICCs of PpIX-TSLT variables showed significant *p*-values and ranged from moderate to good. Although *t*-tests showed significant differences in mitoPO_2 : baseline_ and both mitoDO_2_ variables between iterative measurements, the corresponding effect sizes were low (*d* = 0.32, *d* = 0.27, and *d* = 0.27, respectively). Taken together, the stability of replicate measurements in one session can be seen as moderate to good. We nonetheless recommend multiple measurements in one session. Judging from the LOA and MDD, we can determine that an increase in mitoPO_2 : baseline_ of 35 mmHg or a decrease of 25 mmHg between measurements is probably due to incorrect measurement and the measurement should be repeated. Similarly, absolute changes of 2 mmHg/s for mitoVO_2 : average_ and 5 mmHg/s for mitoDO_2 : average_ could indicate incorrect measurements. All these results are very similar to values determined in our previous study of healthy controls (12).

### Potential Covariates of PpIX-TSLT Measurements

We identified potential covariates of the PpIX-TSLT measurements. We demonstrated a positive association between mitoPO_2 : baseline_ and BIVA vector length, which in turn correlates negatively with hydration status (14, 16). Therefore, more hydrated patients (i.e., with edema), may show lower mitoPO_2_: _baseline_ values. Thus, we recommend considering the presence of edema or conditions with low intravascular oncotic pressure as influence factors when employing the PpIX-TSLT in patients with sepsis. The duration of the 5-ALA plaster application was positively associated with signal quality. One should therefore perform the measurement using a standardized minimum duration of 5-ALA application. Furthermore, room temperature was associated with mitoDO_2 : average_ and signal quality. Signal quality could be improved by performing measurements in a cool environment if possible. The patient's sex posed another covariate. MitoPO_2 : average_ tended to differ between female and male patients. In addition, we found significant sex differences between mitoDO_2 : average_. Both variables showed higher values for females compared to males. This result could indicate the need for sex-specific normal values of PpIX-TSLT variables. Interestingly, neither the status of ventilation nor the dosage of catecholamines showed significant associations with PpIX-TSLT variables.

### Limitations

The generalizability of the results for our secondary objectives are limited by the relatively small number of patients (*n* = 37) and selective exclusion criteria (i.e., exclusion of patients with pre-existing cardiac conditions). Furthermore, in correlative analysis for the identification of potential covariates we did not adjust for multiple testing. Thus, our results need to be confirmed in larger trials. Especially the potential influence of the patient's hydration status on PpIX-TSLT variables should be examined. A direct estimation of total body water using BIA may be useful but is strongly dependent on body weight and height. As measuring body weight accurately in intensive care patients is difficult, we restricted our analyses to the weight-independent BIA variables (BIVA). Due to the fluctuation of physiological variables in critically ill patients, we only analyzed the short-term stability of PpIX-TSLT variables, with iterative measurements within minutes. The long-term stability of PpIX-TSLT variables was not analyzed.

## Conclusion

We conclude that PpIX-TSLT measurements with the COMET are feasible in the critical care setting. Despite the moderate to good stability of the PpIX-TSLT variables using our protocol, we recommend the recording of multiple measurements during one session to increase the reliability of results. The determined limits of agreement and the minimum detectable differences may help to identify potential outlier measurements and additionally improve data quality. Future studies in larger cohorts of critically ill patients are needed to determine the clinical and diagnostic relevance of the PpIX-TSLT using the COMET. Furthermore, healthy controls should be measured to generate normal values for the PpIX-TSLT variables. This study poses a first step toward an evidence-based approach in the assessment of tissue oxygenation in the intensive care unit.

## Data Availability Statement

The datasets generated for this study are available on request to the corresponding author.

## Ethics Statement

The studies involving human participants were reviewed and approved by Ethics Committee of the Friedrich Schiller University Jena. The patients/participants provided their written informed consent to participate in this study.

## Author Contributions

SC, CN, and PB: conception and design of the study. CN, PB, AP, KS, JG, and CS-W: performance of measurements. PB, CN, AP, KS, and JG: clinical data collection. PB, KS, and AP: data analysis and statistical analysis. CN, PB, AP, KS, JG, CS-W, and SC: drafting the manuscript for important intellectual content. CN, CL, PB, and SC: revising the manuscript prior to submission. All authors carefully reviewed and approved the manuscript.

## Conflict of Interest

The authors declare that the research was conducted in the absence of any commercial or financial relationships that could be construed as a potential conflict of interest.

## Abbreviations

5-ALA: 5-aminolevulinic acid
BIA: bioimpedance analysis
BIVA: bioimpedance vector analysis
COMET: Cellular Oxygen Metabolism Monitor
mitoDO_2 : average_: average mitochondrial oxygen delivery
mitoDO_2 : maximum_: maximum mitochondrial oxygen delivery
mitoPO_2 : baseline_: baseline mitochondrial oxygen tension
mitoPO_2 : post_: post-mitochondrial oxygen tension
mitoVO_2 : average_: average mitochondrial oxygen consumption
mitoVO_2 : maximum_: maximum mitochondrial oxygen consumption
PpIX: protoporphyrin IX
PpIX-TSLT: protoporphyrin IX-triplet state lifetime
SEM: standard error of measurement.
