# Supplementary material for: Non-invasive Assessment of Mitochondrial Oxygen Metabolism in the Critically Ill Patient Using the Protoporphyrin IX-Triplet State Lifetime Technique—A Feasibility Study
Source: Front Immunol. 2020 May 7;11:757. doi: 10.3389/fimmu.2020.00757 (PMC7221153; doi:10.3389/fimmu.2020.00757)
Supplement: Supplementary file 1 [file Data_Sheet_1.pdf]

Supplementary Material

Non-Invasive Assessment of Mitochondrial Oxygen Metabolism in the Critically Ill Patient Using the Protoporphyrin IX-Triplet State Lifetime Technique – A Feasibility Study

Charles Neu[1,2,3,a], Philipp Baumbach[1,2,a], Alina K. Plooij[1,2], Kornel Skitek[1,2,], Juliane Götze[1,2], Christian von Loeffelholz[1], Christiane Schmidt-Winter[1,2], Sina M. Coldewey[1,2,3*]

[1] Department of Anesthesiology and Intensive Care Medicine, Jena University Hospital, Jena, Germany

[2] Septomics Research Center, Jena University Hospital, Jena, Germany

[3] Center for Sepsis Control and Care, Jena University Hospital, Jena, Germany

[a] These authors contributed equally to this work.

*** Correspondence:**
Dr. med. Sina M. Coldewey, PhD
Department of Anesthesiology and Intensive Care Medicine
Septomics Research Center
Jena University Hospital
Am Klinikum 1
07747 Jena, Germany
sina.coldewey@med.uni-jena.de

Keywords: sepsis, COMET, mitochondrial dysfunction, critically ill patients, protoporphyrin IX-triplet state lifetime technique, cellular oxygen metabolism, mitochondrial oxygen metabolism, mitochondrial oxygen tension.

Content

S-1 PpIX-TSLT variable distribution information

S-2 Limits of agreement of PpIX-TSLT variables

S-3 Descriptive statistics for BIA variables and potential covariates

S-1 PpIX-TSLT Variable distribution information

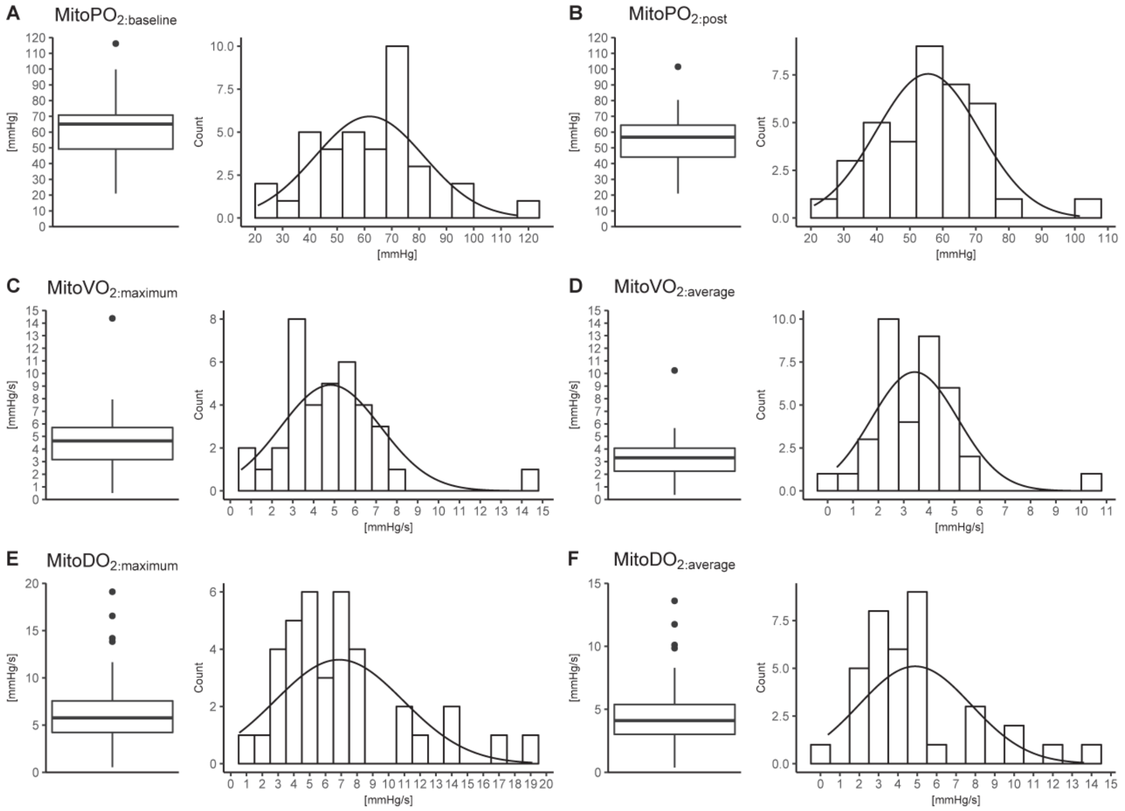

Fig. S-1-1. Distribution of PpIX-TSLT variables. On the left side, boxplots with outlier values (black dots) are displayed. On the right side, histograms with an artificial normal distribution curve are plotted.

Table S-1-1. Distribution parameters and results of the Shapiro-Wilk (S-W) tests of PpIX-TSLT variables. Significant p-values ($p_{S\text{-}W}$) indicate the rejection of the 0-hypothesis that the actual sample was drawn from a normally distributed population. In addition, skewness and kurtosis with standard errors (SE) and excess (kurtosis - 3) are reported.

			Skewness		Kurtosis		
	**S-W**	**$p_{S\text{-}W}$**	**Value**	**SE**	**Value**	**SE**	**Excess**
**MitoPO$_{2: baseline}$**	0.98	.561	0.39	0.39	0.64	0.76	-2.36
**MitoPO$_{2: post}$**	0.97	.521	0.24	0.39	1.11	0.76	-1.89
**MitoVO$_{2: maximum}$**	0.89	**.001**	1.59	0.39	5.99	0.76	2.99
**MitoVO$_{2: maximum}$**†	0.98	.815	-0.20	0.39	-0.50	0.77	-3.50
**MitoVO$_{2: average}$**	0.89	**.001**	1.59	0.39	5.98	0.76	2.98
**MitoVO$_{2: average}$**†	0.98	.817	-0.20	0.39	-0.50	0.77	-3.50
**MitoDO$_{2: maximum}$**	0.89	**.001**	1.31	0.39	1.67	0.76	-1.33
**MitoDO$_{2: maximum}$**†	0.96	.239	0.49	0.41	0.30	0.80	-2.70
**MitoDO$_{2: average}$**	0.89	**.001**	1.31	0.39	1.67	0.76	-1.33
**MitoDO$_{2: average}$**†	0.96	.239	0.49	0.41	0.30	0.80	-2.70

† after removing outliers from the sample (see boxplots in **Fig. S-1-1**)

S-2 Limits of agreement

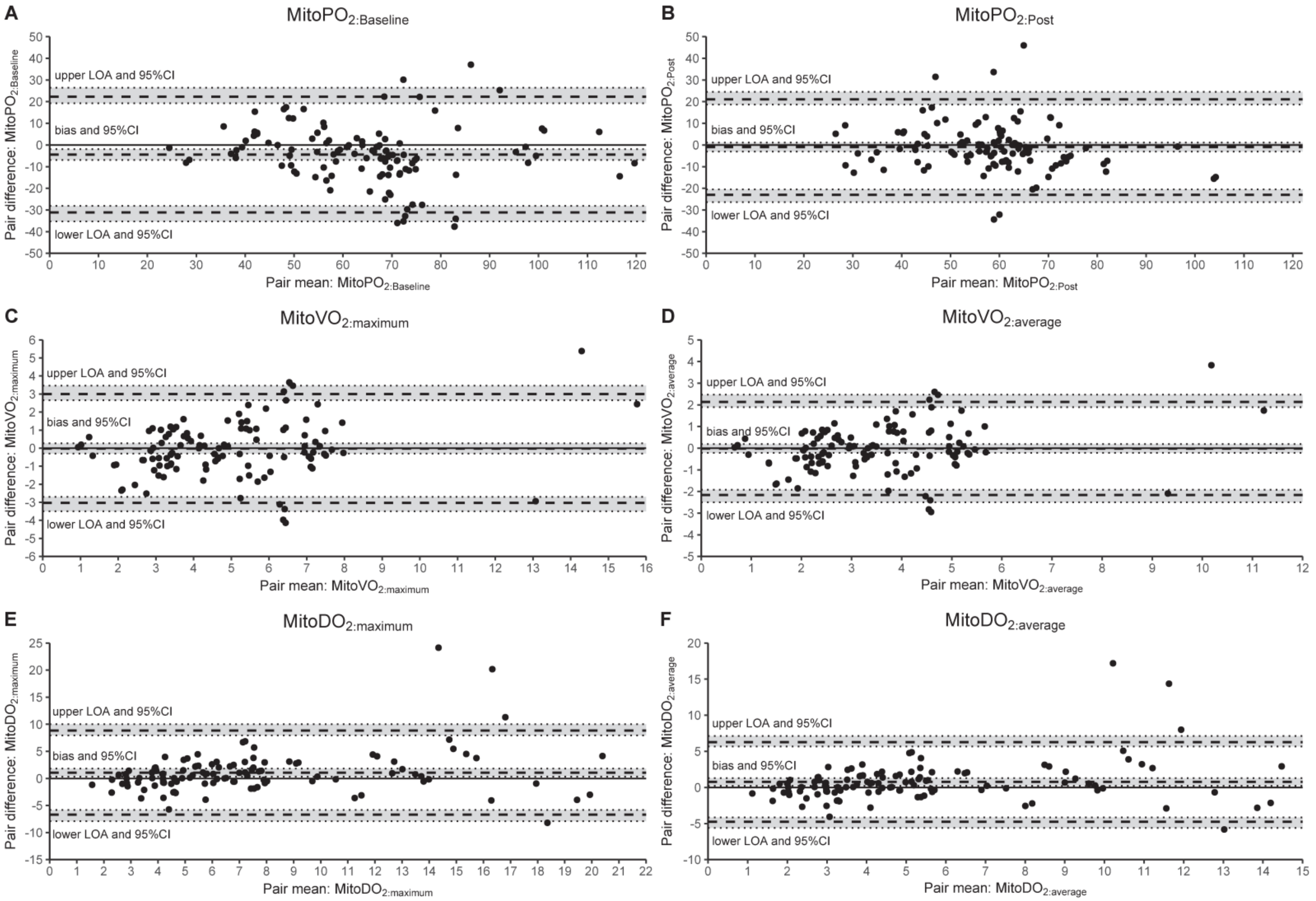

Fig. S-2-1. Bias, upper and lower limits of agreement (LOA) with corresponding 95% confidence intervals (95% CI) for (A) baseline and (B) post mitochondrial oxygen tension ($mitoPO_2$), (C) maximum and (D) average mitochondrial oxygen consumption ($mitoVO_2$), (E) maximum and average mitochondrial oxygen delivery ($mitoDO_2$).

S-3 Descriptive statistics for BIVA variables and potential covariates

Table S-3-1. Median, first and third quartile (Q_1/Q_3) for potential covariates and BIVA variables.

Variable	[Unit]	n	Median	Q_1	Q_3
Duration of ALA application	[h]	36	6.93	6.07	8.39
Signal Quality	[%]	37	48.58	38.38	57.70
Temperature: sensor	[° C]	37	30.90	30.17	31.66
Temperature: room	[° C]	37	23.20	22.90	24.00
Temperature: skin	[° C]	37	33.80	33.10	34.70
Temperature: body	[° C]	37	37.20	36.30	37.70
Goodness of Fit (R^2)		37	0.99	0.98	0.99
Heart rate	[bpm]	37	85.00	74.00	104.00
Blood pressure: systolic	[mmHg]	37	126.00	118.00	146.00
Blood pressure: diastolic	[mmHg]	37	60.00	50.00	65.00
SpO_2	[%]	37	96.00	94.00	98.00
Hemoglobin	[mmol/l]	37	5.40	5.00	6.30
Fluid balance	[l]	37	3.66	1.14	6.32
Fluid balance / body weight	[ml / kg]	37	39.13	15.08	76.96
BIVA: Phase angle	[°]	33	3.50	2.40	4.30
BIVA: Resistance (raw)	[Ω]	33	299.80	265.30	408.80
BIVA: Resistance/Height (R_h)	[Ω/m]	33	1.76	1.51	2.36
BIVA: Reactance (raw)	[Ω]	33	-19.20	-25.30	-11.60
BIVA: Reactance/Height (Xc_h)	[Ω/m]	33	-0.11	-0.15	-0.07
BIVA: vector length (raw)	[Ω]	33	300.02	265.80	409.55
BIVA: vector length ($R/_h$ and $Xc/_h$)	[Ω/m]	33	1.76	1.51	2.36
